# Organic and in-organic fertilizers effects on the performance of tomato (*Solanum lycopersicum*) and cucumber (*Cucumis sativus*) grown on soilless medium

**DOI:** 10.1038/s41598-022-16497-5

**Published:** 2022-07-16

**Authors:** Aruna Olasekan Adekiya, Samuel Olatunde Dahunsi, Jerry Femi Ayeni, Charity Aremu, Christopher Muyiwa Aboyeji, Faith Okunlola, Adeoluwa Emmanuel Oyelami

**Affiliations:** 1 College of Agricultural Sciences, Landmark University, PMB 1001, Omu-Aran, Kwara State Nigeria; 2 Microbiology Programme, College of Agriculture, Engineering and Science, Bowen University, Iwo, Osun State Nigeria; 3 Department of Agricultural & Bio-System Engineering, Landmark University, PMB 1001, Omu-Aran, Kwara State Nigeria; 4 Landmark University SDG 15 (Life on Land Research Group), Omu-Aran, Nigeria

**Keywords:** Ecology, Plant sciences, Environmental sciences

## Abstract

The effects of organic fertilizers, based on *Tithonia diversifolia*, and in-organic fertilizers, based on hydroponics fertilizer, were evaluated on the performance, leaves, and fruit mineral concentrations of tomato (*Solanum lycopersicum*) and cucumber (*Cucumis sativus*) plants grown under soilless medium in a screen house. The treatments comprised six levels of liquid organic fertilizer (5, 15, 25, 35, 45, 55 mL), in-organic fertilizer, and a control. Both organic and in-organic fertilizers increased the growth, yield, leaf nutrient concentration, and the mineral contents of tomato and cucumber fruits in comparison with the control. In-organic fertilizer enhanced the performance and mineral concentrations of tomato and cucumber fruits in comparison with organic fertilizer. However, leaf analyses showed that all the essential elements for both tomato and cucumber crops were within the adequate ranges in the organic fertilizer treatments suggesting that this organic fertilizer can be used as an alternative to the expensive and scarce in-organic fertilizer. For organic fertilizer, the highest yield and mineral contents in fruits were attained at doses of 35 mL and 25 mL for tomato and cucumber plants, respectively. At these doses, the fruit weights of tomato and cucumber were increased by 137 and 198%, respectively, in comparison with the control. For a good yield of tomato and cucumber crop with a high fruit mineral content under the soilless medium of coco peat and rice husk, 35 mL and 25 mL of our tested organic fertilizer are recommended.

## Introduction

Today, Nigeria's population stands at 200 million and is projected to be about 401 million by the year 2050^1^. Sub- Saharan African countries are faced with diverse food challenges^2^. To be able to feed the growing population adequately, food production should approximately double its present output. One of the major constraints to food crop production in the tropics is soil-related problems^3^. In Nigeria, the largest soil order—Alfisol—experiences many unfavorable challenges such as inherently low fertility, soil acidity, poor structure and high susceptibility to crusting, compaction, accelerated erosion, and a decrease in natural resources, among other things, soil diversity reduction^4^. Also, as a result of climate change, the issue of desertification and shorter rainy seasons compounds the problem of food insecurity. Furthermore, growing crops is somewhat difficult in soil-open field agriculture because they require larger areas, many workers, and a large volume of water^5^. Therefore, cropping techniques that bring good control over the environment during the cultivation of a crop give good growth and development of it^6^.

Soilless farming is a cultivation technique by which crops are grown detached from the soil. Crops are grown in a container filled with several possible growing media with nutrients supplied. Soilless cultivation is intensively used in protected agriculture especially for crops during months when field production is not possible, to improve control of the growing environment, and to avoid uncertainties in the soil's water and nutrient status^7^. They are considered important technologies for better water use efficiency as well as high quality and quantity products and they are well adapted to growing vegetable crops such as tomato and cucumber.

Tomato (*Solanum lycopersicum* L.) is a vegetable crop that is cultivated all over the world and is a good source of vitamins A, B6, C, K, and E. It also contains molybdenum, copper, potassium, manganese, and is a good source of dietary fiber. The production of tomatoes using soilless techniques has been reported by several workers^8,9^ and cucumber (*Cucumis sativus* L.) is an important vegetable due to its use as a popular fresh market vegetable in salads and it is one of the most popular members of the Cucurbitaceae family^10^. Cucumbers contain phytonutrients such as flavonoids, lignans, and triterpenes, which have antioxidants, and anti-inflammatory and anti-cancer benefits. The seeds are also a good source of minerals^11^.

The successful production of tomato and cucumber fruits requires an increase in both yield and fruit quality.

The effects of organic and in-organic nutrient sources in soilless techniques on the growth and quality parameters of vegetables had been carried out by many researchers^14^. Phibunwatthanawong and Riddech^15^ found that liquid organic fertilizer had similar growth promotion properties as in-organic fertilizer in the growth of green cos lettuce (*Lactuca sativa* var. *longifolia*). Also, studies on the effects of organic nutrient sources on field vegetable production had been reported^3,16^. However, no research studies have been done on soilless vegetable production in Nigeria with the use of either organic or in-organic nutrient sources. Liedl et al.^17^ found that liquid effluent of digested poultry litter appeared to function as well as a commercial hydroponic fertilizer for tomatoes after balancing the forms of N (NO_3_/NH_4_) and supplementing with Ca(NO_3_)_2_ and MgSO_4_. In Nigeria, no such study existed for soilless cropping.

One of the major ingredients of soilless cropping is inorganic nutrients. The use of inorganic chemical nutrients for agriculture is relatively expensive worldwide^12^. There is an increasing number of screen house farmers who are worried about the cost of hydroponics fertilizers in Nigeria where the rate of exchange of Nigeria Naira to US Dollar is too high and therefore the return rate or benefit/cost ratio of using hydroponics fertilizer is too low. These concerns have started a search for local materials that are readily available (grows along major roads, paths, and on abandoned farmlands in almost every agro-ecological zones of Nigeria) and affordable which could be used as an alternative such as fertilizer obtained from the biomass of Mexican sunflower (*Tithonia diversifolia*).

In addition, during the last decades, the quest for organic products has risen because many people are environmentally conscious and believe that organic products are healthier than conventional products^13^.

Although soilless cropping is done in a controlled environment, reactions of crops in terms of yield and quality of crop to organic nutrients may still be subjected to the environment (tropics, subtropics, or temperate), the quality (chemical properties of different organic material), type of material used and methods for producing organic materials used as organic liquid fertilizer. These aspects need investigation. Therefore, this study was conducted to investigate the effects of organic and inorganic nutrient sources on fruit yield, growth, leaf nutrient concentration, and fruit mineral contents of tomato and cucumber plants grown under the soilless medium.

## Results

### Effects of organic and in-organic fertilizers on growth and yield of tomato and cucumber plants under soilless medium

**Figure 1:**
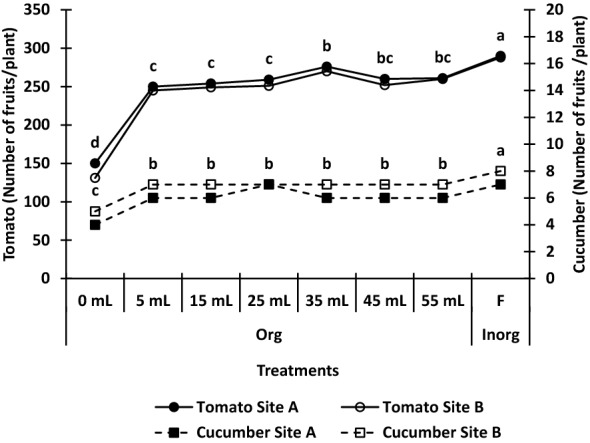
Effect of organic and in-organic fertilizers on number of fruits of tomato and cucumber under soilless medium. Values followed by similar letters under the same graph are not significantly different at p = 0.05 according to Duncan’s multiple range test. *Org* organic fertilizer, *F inorg* in-organic fertilizer.

**Figure 2:**
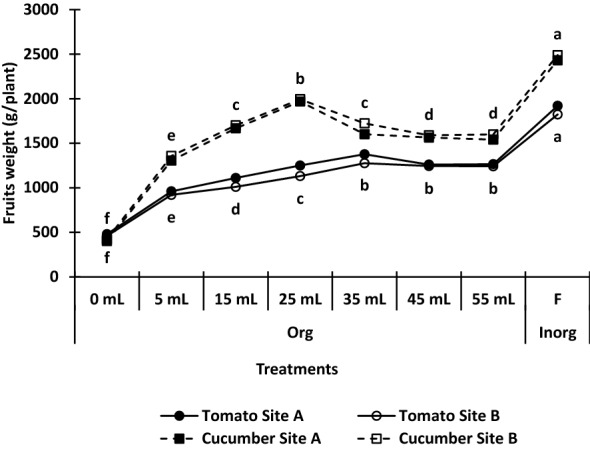
Effect of organic and in-organic fertilizers on fruit weight of tomato and cucumber under soilless medium. Values followed by similar letters under the same graph are not significantly different at p = 0.05 according to Duncan’s multiple range test. *Org* organic fertilizer, *F inorg* in-organic fertilizer.

Figures 1 and 2, and Table 1, respectively, show the results of the effects of organic and in-organic fertilizers on the number of fruits, fruit weight, and growth parameters of tomato and cucumber plants under soilless medium. Both organic and in-organic fertilizers increased the growth (plant height, number of leaves, leaf area, and stem diameter) (Table 1) and the yield (number of fruits and fruit weight) (Figs. 1 and 2) of tomato and cucumber plants significantly compared with the control. For both tomato and cucumber crops, in-organic fertilizer significantly increased growth and yield compared with the organic fertilizer at any dose. Average from both site a and site B, for tomato, in-organic fertilizer increased the yield by 99.04, 76.5, 57.2, 41.1, 49.4, 49.3, and 298.0%, respectively for 5 mL, 15 mL, 25 mL, 35 mL, 45 mL, 55 mL, and the control. Likewise for cucumber, using the average from the two sites, in-organic fertilizer increased the fruit yield of cucumber by 84.8, 46.0, 24.2, 48.01, 56.1, 56.8, and 497.1% respectively for 5 mL, 15 mL, 25 mL, 35 mL, 45 mL, 55 mL, and the control. Also, organic fertilizer increased the growth (plant height, number of leaves, leaf area, and stem diameter) of the two crops up to 55 mL dose.

**Table 1:** Effect of organic and in-organic fertilizers of growth parameters of tomato and cucumber under soilless medium.

Treatment	Plant height (cm)				Number of leaves				Leaf area (cm^2^)				Stem diameter (cm)			
	Tomato		Cucumber		Tomato		Cucumber		Tomato		Cucumber		Tomato		Cucumber	
	A	B	A	B	A	B	A	B	A	B	A	B	A	B	A	B
Control	103.1h	98.2h	97.1h	99.6h	101h	108h	21f	22h	254.1h	251.6h	324.8h	327.7h	2.7f	2.6h	2.5h	2.6h
5 mL	199.8g	187.1g	193.5g	203.4g	128fg	130g	28e	29g	306.5g	301.7g	350.6g	357.1g	3.1e	2.9g	2.9g	3.0g
15 mL	275.6f	267.2f	226.7f	231.1f	135f	139f	38f	34f	366.1f	361.1f	420.2f	428.6f	3.4de	3.3f	3.0f	3.3f
25 mL	298.6e	289.3e	251.8e	260.4e	148e	151e	40ef	42e	389.4e	387.5e	460.1e	466.8e	3.6d	3.5ef	3.3e	3.5e
35 mL	350.1d	345.6d	289.8d	297.6d	159d	160d	46d	47d	401.1d	405.9d	542.1d	546.1d	3.8cd	3.6de	3.9d	3.9d
45 mL	381.5c	380.1c	322.6c	333.2c	170c	173c	51c	53c	445.3c	455.2c	591.6c	599.2c	4.1bc	4.0c	4.4c	4.6c
55 mL	420.6b	419.2b	366.4b	375.1b	199b	201b	61b	65b	486.2b	489.0b	635.4b	648.2b	4.6b	4.4b	4.9b	5.2b
In-organic fertilizer	470.8a	464.3a	394.6a	4.6.2a	231a	243a	69a	72a	530.1a	536.8a	734.7a	745.6a	5.1a	4.9a	5.6a	5.7a
SD ±	12.13	121.9	97.02	123.9	41.27	42.73	16.05	17.36	90.74	94.91	143.5	145.8	0.78	0.76	1.08	1.09

Values followed by similar letters under the same column are not significantly different at p = 0.05 according to Duncan’s multiple range test.

At site A for tomato, 55 mL organic fertilizer increased plant height, number of leaves, leaf area and stem girth relative to the control by 356.0, 97.0, 91.4, and 70.3% respectively, while for site B relative to the control it was 326.9, 86.1, 94.4 and 69.2%, respectively for plant height, number of leaves, leaf area, and stem girth. Also, At site A for cucumber, 55 mL organic fertilizer increased plant height, number of leaves, leaf area and stem girth relative to the control by 277.3, 190.5, 95.4 and 96.0% respectively, while for site B relative to the control it was 276.6, 195.5, 98.1 and 100%, respectively for plant height, number of leaves, leaf area and stem girth. For tomato, organic fertilizer increased yield up to 35 mL level after which there was a decrease. In the same vein, organic fertilizer increased the yield of cucumber up to 25 mL level. At 35 mL for tomato and 25 mL for cucumber), the fruit weight of tomato and cucumber were increased by 137 and 198%, respectively compared with the control. Similarly, inorganic fertilizer increased the fruit weight of tomato and cucumber by 19 and 24%, respectively compared with an organic fertilizer at their optimum level.

### Effects of organic and in-organic fertilizers on leaf nutrient concentrations of tomato and cucumber under soilless medium

Data on the effects of organic and inorganic fertilizers on leaf nutrient concentrations of tomato and cucumber are presented in Table 2. For both tomato and cucumber leaves, nutrient concentration was significantly higher in organic and in-organic treatments compared with the control. Inorganic fertilizer has higher values of N, P, K, Ca, and Mg in tomato leaves compared with organic fertilizer. Organic fertilizer increased nutrient concentration in tomato leaves up to 35 mL level after which there was a decrease at 45 and 55 mL levels.

**Table 2:** Effect of organic and in-organic fertilizers of leaf nutrient concentration of tomato and cucumber under soilless medium (means from sites A & B).

Treatment	N (%)		P (%)		K (%)		Ca (%)		Mg (%)	
	Tomato	Cucumber	Tomato	Cucumber	Tomato	Cucumber	Tomato	Cucumber	Tomato	Cucumber
Control	3.81g	2.21g	0.44f	0.24f	4.51f	2.81e	3.92g	1.82f	0.44f	0.51f
5 mL	5.34f	3.67f	1.70e	0.50c	8.21e	3.01cd	4.45f	2.22d	0.50e	1.12c
15 mL	5.91e	4.05e	2.20d	0.57b	9.16d	3.22bc	5.12e	2.61b	0.55d	1.31ab
25 mL	6.06d	4.55d	3.10c	0.65a	9.80bc	3.84a	6.02c	2.82a	0.60c	1.41a
35 mL	10.13c	4.43c	4.20b	0.60a	10.13b	3.61ab	6.42ab	2.71ab	0.86b	1.43a
45 mL	11.14b	4.92b	3.00c	0.43de	9.65bc	3.41bc	5.63d	2.44c	0.86b	0.84e
55 mL	11.98b	5.74a	2.10d	0.40e	9.44cd	3.22bc	5.76d	2.01e	0.84b	0.81e
In-organic fertilizer	18.56a	3.54d	4.70a	0.56d	12.30a	3.01cd	6.67a	2.03e	0.91a	0.90d
SD ±	4.84	1.05	1.37	0.13	2.20	0.34	0.95	0.36	0.19	0.32

Values followed by similar letters under the same column are not significantly different at p = 0.05 according to Duncan’s multiple range test.

For cucumber, leaf N, P, K, Ca, and Mg with organic fertilizer were increased from 5 to 25 mL application level after which there was a reduction. There were no significant differences between 5 mL, 55 mL, and inorganic fertilizer for N and K in cucumber, also all levels of organic fertilizer produced significantly higher values of P and Mg in cucumber leaves compared with inorganic fertilizer except those for 45 and 55 mL levels.

### Effects of organic and in-organic fertilizers on mineral contents of tomato and cucumber fruits under soilless medium

Results of the effects of organic and inorganic fertilizers on mineral contents of tomato and cucumber fruits under soilless conditions are presented in Table 3. In both tomato and cucumber crops, mineral contents of the fruits were significantly higher under organic and inorganic fertilizers compared with the control. Mineral contents were also higher in inorganic treatment compared with organic treatments. Tomato has significantly higher mineral content (P, K, Ca, Mg and Fe) at 35 mL level of organic fertilizer. Similarly, cucumber has its optimum mineral nutrient concentration at 25 mL of organic fertilizer. The value of N in both crops increased by up to 55 mL.

**Table 3:** Effect of organic and in-organic fertilizers of on mineral contents of tomato and cucumber fruits under soilless medium (means from sites A & B).

Treatment	N (mg kg^−1^)		P (mg kg^−1^)		K (mg kg^−1^)		Ca (mg kg^−1^)		Mg (mg kg^−1^)		Fe (mg kg^−1^)	
	Tomato	Cucumber	Tomato	Cucumber	Tomato	Cucumber	Tomato	Cucumber	Tomato	Cucumber	Tomato	Cucumber
Control	20.3h	30.4h	116.2g	92.3e	2.4g	2.9e	90.1f	50.1e	33.4g	22.3f	6.6g	2.35e
5 mL	26.8g	34.4g	146.3f	108.1d	10.5f	3.3bc	106.3e	68.2c	37.8f	29.2de	8.9f	3.0d
15 mL	31.9f	36.8f	164.3e	126.0c	17.8d	3.4b	154.1d	78.6b	42.4e	38.3c	10.5e	3.5c
25 mL	41.2e	41.0e	188.8c	163.0b	24.6d	3.5b	176.8c	79.6b	49.6c	43.1b	12.7d	3.9b
35 mL	55.2d	46.3d	207.8b	105.1d	30.1b	3.0de	188.2b	65.1d	56.4b	38.2c	20.6b	3.1d
45 mL	60.4c	51.5c	178.9c	94.2e	25.8cd	3.2cd	176.6c	68.2cd	49.8c	22.1f	20.1bc	2.45e
55 mL	68.1b	56.1b	176.6d	100.1d	26.4c	3.2cd	174.3c	64.6d	48.4cd	27.7e	19.8c	2.20f
Inorganic fertilizer	80.4a	65.1a	246.7a	168.2a	36.4a	5.8a	196.3a	90.6a	60.1a	73.1a	29.7a	7.4a
SD ±	21.31	11.82	39.16	30.23	11.00	0.93	38.99	12.20	8.99	16.59	7.74	1.68

Values followed by similar letters under the same column are not significantly different at p = 0.05 according to Duncan’s multiple range test.

## Discussion

The increase in growth and yield of tomato and cucumber crops in the present study due to the application of organic and inorganic fertilizers was a result of the increase in nutrient status of the substrate because of the applied nutrients leading to absorption, hence significant improvement in growth, yield leaf nutrient concentration and fruit mineral levels of tomato and cucumber crops.

The results are in agreement with that of Mowa et al.^18^ that organic plant sources can certainly supply adequate nutrients for good growth and yield of crops under soilless cropping. The improved growth as the rates of organic fertilizer increase can be adduced to increased nutrient supply, especially N that is needed for vegetable growth of crops.

For both tomato and cucumber crops, the number of fruits per plant was higher in inorganic fertilizer compared with organic fertilizers. Tonfack et al.^19^ had earlier reported an increase in fruit per plant where in-organic fertilizer was used relative to organic fertilization.

Organic fertilizer reduced the growth, yield, and mineral contents of tomato and cucumber fruits compared with inorganic fertilizer. This was due to low availability, slow release, and low uptake of organic nutrients by the crops compared with the inorganic fertilizer. Mowa et al.^18^ reported that organic fertilizers are unsuitable for planting growth because N in organic fertilizers is predominantly organic, hence unusable by plants. Unlike inorganic fertilizer that releases nutrients upon application/fertigation, organic fertilizer has to mineralize first before being available to crops. Baghdadi et al.^20^ reported that the number of nutrients which contacts directly with the plant roots in organic fertilized crops is rather small within the overall nutrient demand hence reducing growth and yield compared with inorganic fertilizer. Another reason for the better growth and yield of crops under inorganic compared to organic fertilizer was due to differences in nitrate and other nutrient levels between the inorganic nutrient solution and organic nutrient solution.

In the current study, inorganic fertilizer had NH_4_-N (48.7 mg L^−1^ N), NO_3_-N (173.4 mg L^−1^ N), PO_4_-P (107.8 mg L^−1^ P), K (173.8 mg L^−1^), Ca (158.5 mg L^−1^), Mg (80 mg L^−1^) and SO_4_-S (106.7 mg L^−1^ S) compared with organic nutrient with 48.5, 3.41, 4.5, 8.0, 100.0 and 84.0 mg L^−1^ respectively for N, P, K, Ca, Mg and SO_4_ (Table 4). Likewise, results showed that growth parameters of tomato and cucumber increased with the increase of organic nutrient levels up to 55 mL level. In contrast, the yield was increased with tomato and cucumber up to 35, and 25 mL respectively can testify that in the experimental condition, the 35 mL was over optimal for the yield of fruits of tomato and 25 mL was for cucumber fruits. The results corroborated that of Simpson^21^ who found that tomato fruit yields were depressed as N rate was increased by producing excessive luxuriant vegetative parts of the plant at the expense of reproductive growth. Similarly, excessive fertilization of N fertilizer has been reported to cause decreases in the commercial yield of cucumber^22^. Therefore, Nitrogen fertilization must be carefully managed to attain a high marketable yields while minimizing the adverse effects of excessive vine growth. For organic fertilizer, this high marketable yield was attained for tomato at 35 mL and 25 mL for cucumber. It shows that tomato requires more nutrients than cucumber. This fact is evident in the higher nutrient concentrations of tomatoes compared to that of cucumbers (Table 2).

**Table 4:** Chemical properties of liquid organic fertilizer used for the experiment.

Element	Value
Nitrogen (mg L^−1^)	48.50 ± 1.10
Phosphorous (mg L^−1^)	3.41 ± 0.06
Potassium (mg L^−1^)	4.50 ± 0.05
Calcium (mg L^−1^)	8.00 ± 0.60
Iron (mg L^−1^)	5.40 ± 0.50
Manganese (mg L^−1^)	0.02 ± 0.001
Zinc (mg L^−1^)	22.50 ± 0.80
Aluminum (mg L^−1^)	0.59 ± 0.02
Nitrite (mg L^−1^)	2.80 ± 0.04
Sulphate (mg L^−1^)	84.00 ± 2.10
Phosphate (mg L^−1^)	99.70 ± 2.20
pH	7.90 ± 0.50
Nickel (mg L^−1^)	5.00 ± 0.40
Copper (mg L^−1^)	3.10 ± 0.03
Boron (mg L^−1^)	4.80 ± 0.05
Magnesium (mg L^−1^)	100.00 ± 3.2

Leaf nutrient concentration was significantly higher in organic and inorganic treatments compared with the control. The response of leaf nutrient concentrations of tomato and cucumber leaves to organic and inorganic fertilizer was consistent with the values of nutrients recorded for organic fertilizer (Table 4) and that for inorganic fertilizer. There was increased nutrient availability in the substrate leading to increased uptake by tomato and cucumber plants. Leaf nutrient concentrations of inorganic fertilizer under tomato crops were improved compared with those of the organic fertilizers because of the quick and easy absorption of nutrients in the inorganic treatments. In cucumber crops, organic fertilizer improved leaf nutrient concentration compared with inorganic fertilizer. This was due to the fact that some of the nutrient elements may have been converted to assimilate (fruits). In the present experiment, inorganic fertilizer has a higher yield compared with organic fertilizer for cucumber. Furthermore, for this experiment, leaf analyses showed that all the essential elements for both tomato and cucumber crops were within the adequate ranges in the organic and inorganic fertilizer treatments as suggested by^23^ for greenhouse-grown tomato (4.5% N, 0.56% P, 5.72% K, 4.4% Ca and 0.50% Mg) and de Kreij et al.^24^ for cucumber (4.2% N, 0.6 P, 3.2% K, 2.2 Ca and 0.40% Mg) at optimum levels. It shows that the amounts of other nutrients required for tomato plant growth were sufficient in the organic nutrient solution to result in similar growth patterns as those from the inorganic fertilizer. In this experiment, the use of *Tithonia* biomass as fertilizer in tomato and cucumber production was carried out with virtually no cost relative to the expensive inorganic fertilizer. It suggested that this organic fertilizer can be used as an alternative for the expensive and scarce inorganic fertilizer in developing countries like Nigeria. Chang et al.^25^ also found that organic fertilizer produced from pea and rice hull compost can successfully replace chemical fertilizer for cut flower production of *Anthurium andreanum*.

Except for N, the mineral contents of both tomato and cucumber fruits increased up to 35 mL and 25 mL respectively, due to nutrient imbalance after the optimum level of nutrients^3^. At a high N rate from the excessive rate of nutrients, crops (tomato and cucumber) produced many leaves with dark green flourishing big growth and abnormal cell as a result of lack of other elements such as Mg, K, and Ca. The fruits have high water content but fewer flavors and become watery^3^. Roper^26^ reported that nutrients in the fruits declined with an increase in nutrient applications.

## Conclusion

The results of this study revealed that both organic and inorganic fertilizers increased the growth, yield, leaf nutrient concentration, and the mineral contents of tomato and cucumber fruits compared with the control. Although organic fertilizer reduced the growth, yield, and mineral contents of tomato and cucumber fruits compared with inorganic fertilizer (which was due to low availability, slow-release, and low uptake of organic nutrients by the crops compared with the inorganic fertilizer), leaf analyses showed that all the essential elements for both tomato and cucumber crops were within the adequate ranges in the organic fertilizer treatments suggesting that this organic fertilizer can be used as an alternative for the expensive and scarce inorganic fertilizer. Also, for organic fertilizer in this experiment, the highest yield and mineral contents were attained for a tomato at 35 mL and 25 mL for cucumber. It shows that this is the optimum level for these crops, and that tomato requires more nutrients than cucumber. Therefore, for a good yield of tomato and cucumber with high mineral content under the soilless medium of coco peat and rice husk, 35 mL and 25 mL are recommended for tomato and cucumber respectively.

## Material and methods

### Growth conditions and plant materials

Two experiments were conducted concurrently (sites A and B) in the same screen house in 2019 between the months of May and July at the Landmark University Greenhouse and Hydroponic Technology Center, a section of the Teaching and Research Farm of the University in Omu-Aran, Kwara State Nigeria. Experiment at site B was conducted simultaneously as A so as to validate the results of experiment A. Landmark University lies within Latitude 8° 7′ 26.21388″ and 5° 5′ 0.1788″. Both experiments (A & B) involved tomato (*Solanum lycopersicum* L. variety cherry) and cucumber (*Cucumis sativus* L. variety marketer) crops. For each crop, seeds were sown into a separate seed tray filled with coco peat (Coco peat, SRIMATHI EXPORT, INDIA). Cocopeat is the mesocarp tissue or husk after the grinding of coconut fruit. It has a lightweight and high water and nutrient holding capacities, it has an acceptable pH, electrical conductivity, and other chemical attributes^27^. Rice husk is the by-product of rice after milling. The rice husk used was collected from the rice processing mill of Landmark University. Rice husk is a highly porous and light weighted material with a very high specific area^28^.

Two sets of seed trays (one for organic and another for inorganic fertilizers) were used each for tomato and cucumber crops in the nursery. Both were raised in the nursery for two weeks before transplanting. Black grow bags (30 × 17 cm) filled with a coco peat/rice husk (1:4 ratio by volume) mixture with a weight of about 10 kg were arranged in a screen house. Both the nursery and establishment of crop proper take place in a screen house. The screen house has a galvanized iron as the frame, a UV covering on top, side net for screening insect pests the floor fairly covered with granite. Temperature and relative humidity within the screen house during the period of the experiment was monitored using a Thermograph and a Barograph, and they were at an average of 31 °C and 75%, respectively.

The grow bags were randomly placed in the screen house for the unbiased application of amendments. For both tomato and cucumber crops, the treatment comprised of six (6) levels of liquid organic fertilizer (5, 15, 25, 35, 45, 55 mL), in-organic fertilizer, and a control (ordinary borehole water). Levels of organic fertilizers were selected based on the recommendation of 20 mL of liquid organic fertilizer by^29^. The eight (8) treatments both for tomato and cucumber were arranged in a Completely Randomized Design replicated three times. One healthy plant was maintained per grow bag and four grow bags represent a treatment and there were 32 plants per block each for tomato and cucumber. For both crops, the experiment lasted for 90 days.

### Organic and in-organic nutrient solutions

The liquid organic fertilizer used was obtained from the biomass of Mexican sunflower (*Tithonia diversifolia*). Fresh biomass (mainly leaves and stems) of the plant was collected from the Teaching and Research Farms of Landmark University, Nigeria. After rinsing, they were cut with a sterile knife into pieces of ≤ 1 cm size. A sample was taken for initial physicochemical analyses by grinding in a sterile mortal, diluted with sterile water and analyzed. The biomass was then soaked in sterile water inside a clean container, and allowed to ferment spontaneously for a period of 14 days. During the fermentation, samples were taken every 4 days for microbial analyses of the major players during the fermentation. At the end of fermentation, the mixture was separated using a sieve of mesh size ≤ 2 mm. The liquid portion was then refrigerated prior to the planting regime while another sample was taken to ascertain the physicochemical and microbial qualities of the produced liquid fertilizer. The chemical analysis is presented in Table 4. For inorganic fertilizer, Water soluble fertilizers employed in hydroponics were used (Hydroponics fertilizer, Anmol chemicals, India); calcium nitrate 650 mg L^−1^, potassium nitrate 450 mg L^−1^, magnesium 400 mg L^−1^, chelate 20 mg L^−1^, mono-ammonium phosphate 400 mg L^−1^. The electrical conductivity (EC) of the solution was 1.9 dS m^-1^.

### Irrigation and fertigation

The tomato and cucumber plants were fertigated morning and evening daily for one hour on each occasion according to the treatments. Preparation of the nutrient solution was with borehole water and was supplied to plants by an online pressure drip irrigation system set at 2.0 L h^-1^ using an arrowhead on each tomato and cucumber plant. Different tanks (250 L) were installed according to the various treatments making a total of 8 tanks. The organic fertilizer was diluted according to the various treatments equivalent to 1.25, 3.75, 6.25, 8.75, 11.25, and 13.75 L per 250 L of water respectively for 5, 15, 25, 35, 45, and 55 mL treatments. The nutrient solutions were refilled when the consumption is less than 20% of the initial volume (250 L) in the tank. One day per week, crops were irrigated with ordinary water to wash out pipes and prevent deposits of salts. The same concentration of nutrient was used from transplanting to the termination of the study for both tomato and cucumber crops, however, at the flowering of the crops, the volume of fertigation was increased to 3.0 L h^-1^ to be able to cope with the size of the plants.

### Trellising, pest and diseases control

For both tomato and cucumber crops, plant vines were supported by twisting them around a wire that is- attached to the roof of the screen house and 2 m from the ground. Lateral outgrowths were cut off every week to ensure a sturdy single stem. Pests and diseases were scouted every day. Whiteflies, aphids, and other insects were controlled with orizon (Producer, location of producer) (active ingredient, acetamiprid, and abamectin) using 0.133% v/v. Fungi were controlled using ridomil gold (Producer, Location of producer) at 2% w/v.

### Determination of growth and yield of tomato and cucumber

Three tomato and cucumber plants were randomly selected for each treatment for the determination of growth parameters (plant height, leaf area, number of leaves per plant, and stem diameter) at mid the flowering stage of tomato and cucumber plants.The leaf area of tomato was calculated using the model (A = KL2) developed by Lyon^30^, where L = Length of tomato leaf, K = constant which is 0.1551, and A = leaf area of tomato. Similarly, the leaf area of cucumber was calculated using A = 0.88LW – 4.27, where L = cucumber leaf length and W = cucumber leaf width, A = leaf area of cucumber^31^.

Tomato fruits were ready for harvest from 65 days after transplanting, harvestings were done twice every week (Mondays and Fridays) for up to 85 days after transplanting. Similarly, harvesting of cucumber fruits started 35 days after transplanting and harvestings were also done twice a week (Mondays and Fridays), harvesting was carried out till 60 days after transplanting. Tomato and cucumber fruit yields were counted and weighed at each harvest.

### Analysis of tomato and cucumber leaves and fruits

At the 50% flowering stage of tomato and cucumber plants, ten leaf samples were collected from each treatment. The leaf samples were oven-dried at 75 °C for 24 h and thereafter grounded. The grounded samples were later analyzed for nitrogen (N), phosphorous (P), potassium (K), calcium (Ca), and magnesium (Mg) content using the method of described by^32^. At harvest, four matured tomato and cucumber fruits of uniform size were selected per treatment, and their nutrient compositions were determined using the method of^33^.

### Statistical analysis

All data collected on the growth, yield, leaf, and fruit nutrient contents of tomato and cucumber were subjected to analysis of variance (ANOVA). The SPSS V 21.0 (New York, USA) software was used to perform ANOVA and Duncan’s multiple range test (DMRT) was used to compare means at a 5% probability level.

### Ethical approval

I confirm that all the research meets ethical guidelines and adheres to the legal requirements of the study country.

### Compliance with international, national and/or institutional guidelines

Experimental research (either cultivated or wild), comply with relevant institutional, national, and international guidelines and legislation. Experimental studies were carried out in accordance with relevant institutional, national or international guidelines or regulation.

## Data Availability

All datasets generated and/or analyzed during the current study are included in this article.
